# Variability in bat morphology is influenced by temperature and forest cover and their interactions

**DOI:** 10.1002/ece3.9695

**Published:** 2023-01-29

**Authors:** Heather Wood, Sara A. O. Cousins

**Affiliations:** ^1^ Landscape, Environment & Geomatics, Department of Physical Geography Stockholm University Stockholm Sweden; ^2^ Bolin Centre for Climate Research Stockholm University Stockholm Sweden

**Keywords:** bats, body size, functional traits, land‐use, morphology, temperature

## Abstract

Multiple climatic and landscape drivers have been linked to variations in bat body size and wing functional traits. Most previous studies used proxies rather than actual climate and land‐use data, and their interactions are rarely explored. We investigate whether higher summer average temperatures are driving decreasing bat body size as predicted by Bergmann's rule or increasing appendage size as per Allen's rule. We also explore whether temperature or resource availability (namely forest cover) is responsible for changes in wing functional traits. Using land‐use data from historical maps and national statistics combined with climatic data, we assessed the effect of temperature and resource availability on bat morphology. We used 464 museum specimens of three bat species (*Eptesicus nilssonii*, *Pipistrellus pygmaeus*, and *Plecotus auritus*), spanning 180 years, across a 1200 km latitudinal gradient. We found no evidence of higher summer average temperatures driving decreases in body size in bats. Jaw sizes of *P. auritus* and *P. pygmaeus* changed over time but in different directions. The geographical variation of forest cover was also related to differences in wing functional traits in two species. Crucially, there was a significant antagonistic interactive effect of forest and temperature on tip index in *P. pygmaeus* whereby above 14.5°C the relationship between forest and tip index actually reversed. This could indicate that higher temperatures promote more pointed wings, which may provide energetic benefits. Our results show the importance of including both climatic and land‐use variables when assessing trends in bat morphology and exploring interactions. Encouragingly, all three species have shown an ability to adapt their body size and functional traits to different conditions, and it could demonstrate their potential to overcome future negative impacts of climate and land‐use change.

## Graphical abstract

To decouple the effects of global drivers on bat morphology, we used a combination of high spatial and temporal resolution climate and land‐use data to assess how bat cranial size and wing functional traits respond to changing temperatures and forest cover over 180 years along a 1200 km latitudinal gradient. We found the effects of temperature and land use on morphology, but more importantly, they interacted antagonistically causing variation in bat morphology in different directions.

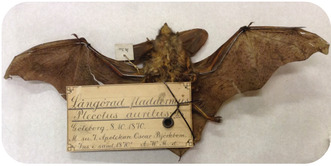

## 1 INTRODUCTION

It is undisputable that environmental change is responsible for wide‐ranging impacts on our global biodiversity (IPBES, 2019). Some of these impacts are well documented such as changes to species distributions and ranges (Sirami et al., 2017), phenology (Visser & Both, 2005), diversity (Newbold et al., 2015), and extinctions (Brook et al., 2008); whereas others have been less in focus, such as morphological change. Species need to track their environmental envelope either by dispersing or adapting and emerging evidence shows that species may actually adapt their morphology, for example, body size and functional traits, as a response to climate and land‐use changes (Gardner et al., 2009; Ryding et al., 2021; Vanhooydonck et al., 2009; Yom‐tov et al., 2008).

Historically, biogeographical rules such as Bergmann's (1848) and Allen's (1877) rules have been used to explain spatial variation in morphology due to exposure to different climatic conditions. The need for species thermoregulation is at the centre of these two rules with Bergmann's predicting increased body size at higher altitudes and latitudes due to decreasing temperatures necessitating a smaller surface area to volume ratio to retain heat. Whereas Allen's Rule states that animals have larger appendages, such as wings, beaks, and ears, in relation to body size in warmer environments due to improved heat loss via the larger surface area of these extremities.

Although there is limited research on the effect of land‐use or resource availability changes on morphology, it is widely known that wing functional traits of bats, birds, and insects are based on their ecological requirements and that wing shape correlates with habitat preferences. Due to the high energy demands of flight, organisms require wings that allow for efficient flight (Norberg, 1994). Those with long, narrow wings are open habitat specialists and this wing structure reduces energy expenditure. By contrast, short, broad wings increase maneuverability for flight in complex forest environments (Norberg & Rayner, 1987). The shape of wing tips is also important, with more pointed wings enabling faster flight (Findley et al., 1972) and migrant birds with more pointed tips fly more efficiently at higher speeds (Bowlin & Wikelski, 2008). Recently it has been shown that birds and insects have adapted wing morphology due to environmental stressors (Brown & Brown, 2013; Desrochers, 2010). Habitat types could act as a filter (Zobel et al., 1998) favoring organisms more adapted in their wing shape and flight maneuverability. For example, in fragmented forest landscapes, they may need to travel over more open habitats, which could favor long, narrow wings. Furthermore, considerable changes in forest cover and structure have occurred in Europe with a shift to increasing forest monocultures (Bradshaw, 2004) and denser tree stands (Jansson, 2011), in part due to the cessation of forest grazing (Jensen, 2011). This increased forest density could favor more maneuverable species with shorter, broader, and less pointed wings and may have forced species to adapt their behavior in response to the extensive changes to their preferred habitats. Hence the widespread land‐use changes, which have occurred across Europe in the last 100–150 years have dramatically altered resource availability and could potentially drive various temporal changes in wing morphology within affected species.

Past research on morphological change has focused on birds and small rodents, possibly due to the overrepresentation of these taxa in museum collections—a common source of morphological data (Holmes et al., 2016). The short generation times of these species' groups may allow the detection of phenotypic changes over relatively short time periods. However, few studies have focused on bats (Salinas‐Ramos, Agnelli, Bosso, Ancillotto, & Russo, 2021; Salinas‐Ramos, Agnelli, Bosso, Ancillotto, Sánchez‐Cordero, et al., 2021; Snell‐rood & Wick, 2013; Tomassini et al., 2014; Yue et al., 2020). These taxa are known for their longevity in relation to their body size (Munshi‐south & Wilkinson, 2010) with some European bats living for 20–40 years and this may limit the ability to detect change over relatively short time periods. Bats are known, however, to be sensitive bioindicators (Jones et al., 2009), which could increase the detectability of environmental change effects. Previous studies on morphological changes in bats confirm effects are detectable over relatively short time frames, where crania of bats changed in response to land‐use modification over only 100 years (Snell‐rood & Wick, 2013; Tomassini et al., 2014) and forearms increased between 65 and 150 years (Salinas‐Ramos, Agnelli, Bosso, Ancillotto, & Russo, 2021; Yue et al., 2020). This demonstrates the suitability of bats as a study group on morphological variability due to climate and landscape change.

So far, temperature and land‐use‐mediated effects on morphology have been detected mostly using time as a proxy to represent temperature and land‐use variables (Desrochers, 2010; Gardner et al., 2009; Tomassini et al., 2014). In turn, interactions of co‐occurring drivers are rarely explored. Although some studies used measured temperature (Salewski et al., 2014; Yom‐tov et al., 2008, 2010) or historic land‐use change data (Yue et al., 2020) to test their hypotheses, most other studies do not include these important variables. Today, there are many sources of global climate data available (Harris et al., 2014; Morice et al., 2012), and these can easily be utilized in analyses. However, obtaining accurate historic land‐use data is a greater challenge. Historic maps are an excellent resource and now with the possibility of rapid digitization, extracting this valuable data has become simpler (Auffret et al., 2017), and if maps are unavailable or lack temporal resolution, they can be supplemented with historic national or regional statistics on land use (Pasanen‐Mortensen et al., 2017).

To decouple the effects of global drivers, we used a combination of high spatial and temporal resolution temperature and land‐use data to assess how bat cranial size and wing functional traits respond to temperature and resource availability (using forest cover as a proxy) over 180 years along a 1200 km latitudinal gradient. We hypothesized that: (1) body size would respond to summer average temperature (2) wing shape would vary in relation to forest cover (3) temperature and forest would have an interactive effect on wing shape. We predicted that: (1) smaller body size and larger appendage size would be positively correlated to summer average temperature, as per Bergmann's and Allen's rules (2) shorter, broader, and less‐pointed wings would be favored in more forested landscapes (3) temperature and forest cover would interact antagonistically to drive wing shape in opposite directions.

## 2 METHODS

All available, intact, Swedish museum specimens with complete collection metadata of three bat species, *Eptesicus nilssonii*, *Pipistrellus pygmaeus*, and *Plecotus auritus* were measured (*n* = 464). These species were chosen because they represent slightly different foraging guilds with *E. nilssonii* tending to forage in more open areas; *P. pygmaeus* preferring the interface between open and forest areas and *P. auritus* more often associated with forested areas (De Jong et al., 2020). We combined the measurements of these bats with historic land‐use data extracted from historical maps and Swedish national statistics on Agriculture and Livestock (Statistiska centralbyrån, 1868, 1869, 1900, 1920, 1944, 1952) and summer average temperature data extracted from the CRU database (Harris et al., 2014) and weather stations (SMHI, 2019), to explore the drivers of size and functional trait variation over 180 years.

### 2.1 Bat morphology

**FIGURE 1:**
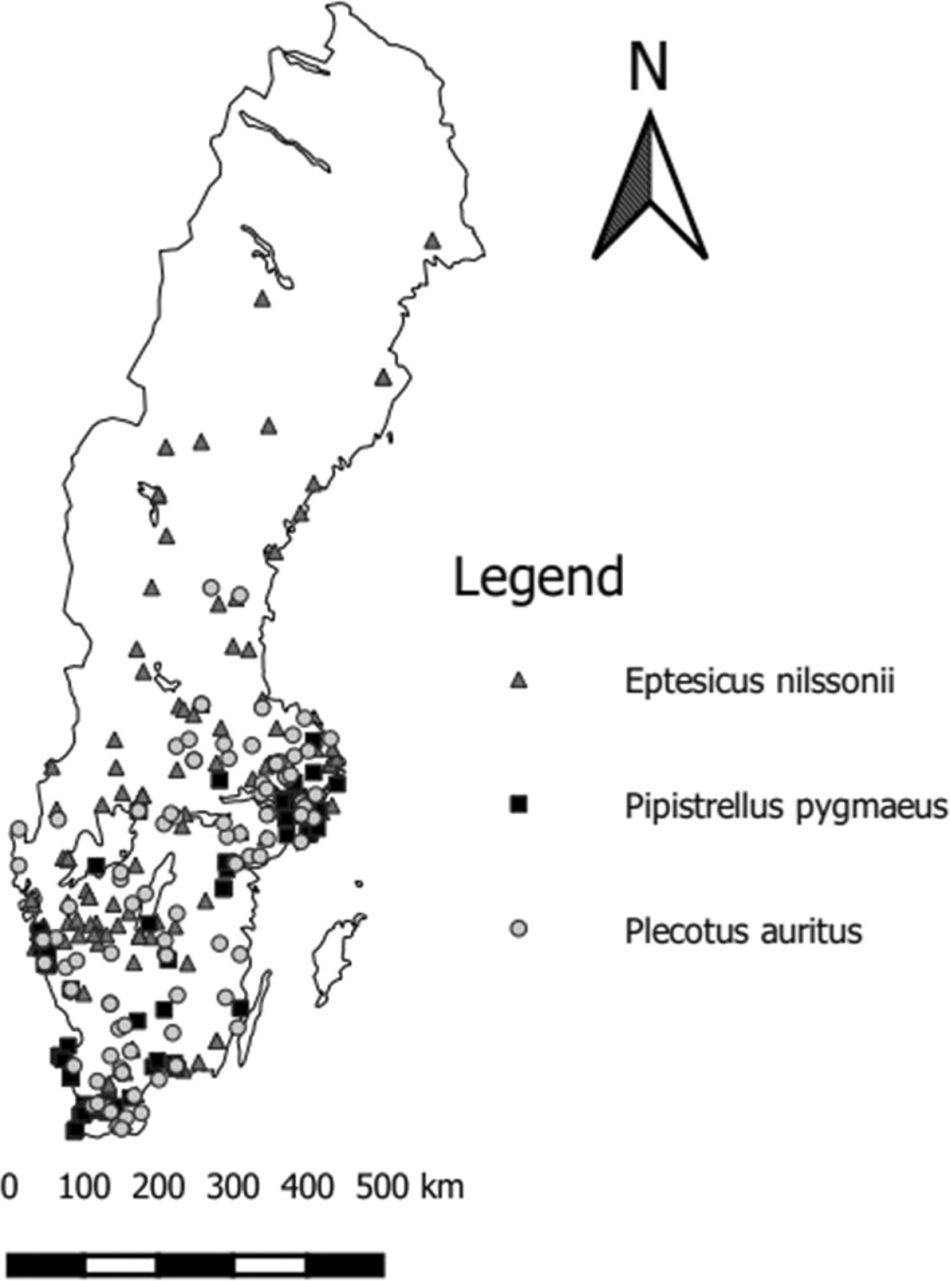
Distribution of museum specimens (*n* = 464) of three bat species, *Eptesicus nilssonii* (*n* = 144), *Pipistrellus pygmaeus* (*n* = 192), and *Plecotus auritus* (*n* = 128), measured within this study. The specimens were collected between latitudes 55.38N and 66.07N.

All specimens were collected in Sweden with the majority located in southern and central regions (Figure 1). Specimens ranged in latitude from 55.38°N to 66.07°N and this represents a gradient of approximately 1200 km. We measured specimens from the Swedish Museum of Natural History (S), Gothenburg Natural History Museum (GNHM), Lund Museum of Zoology (MZLU), Uppsala Evolution Museum, and the National Museum of Natural History, Smithsonian Institute (NMNH). Both crania and wings of bats were measured. All crania and jaw parameters are morphological measurements of body size whilst wing area index and forearm length are measurements of wing size; forearm length is not considered within this study as a proxy for total body size as this has been shown to be a poor indicator of body mass within species (McGuire et al., 2018). Forearm length can instead be considered as a functional trait along with the aspect ratio index and tip index. These functional traits are related to flight maneuverability and speed where the high aspect ratio index and tip index are related to long, narrow, wings that are adapted for fast flight in open areas. Wings with the low aspect ratio index and tip index are shorter and broader and are adapted for high maneuverability in forest environments (Findley et al., 1972; Norberg & Rayner, 1987).

**FIGURE A1:**
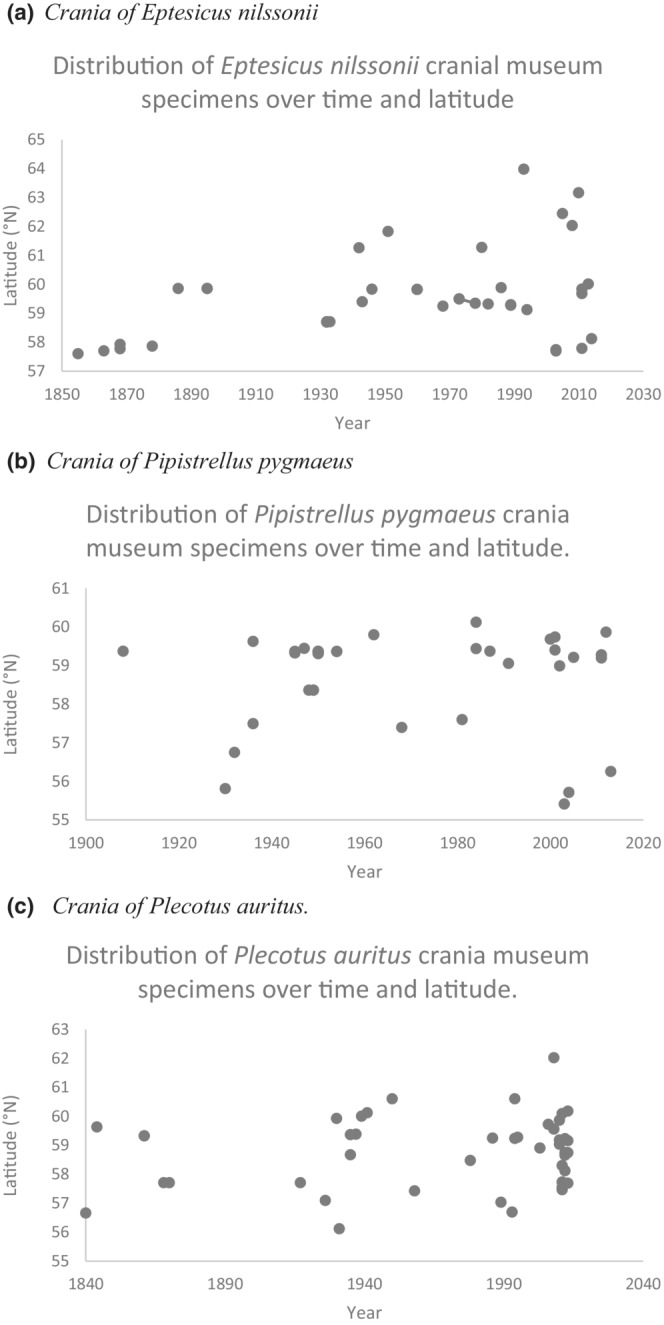
Spatial and temporal distribution of cranial museum specimen collections showing the date of collection against the latitude of collection place in Sweden for three bat species (a–c).

**FIGURE A2:**
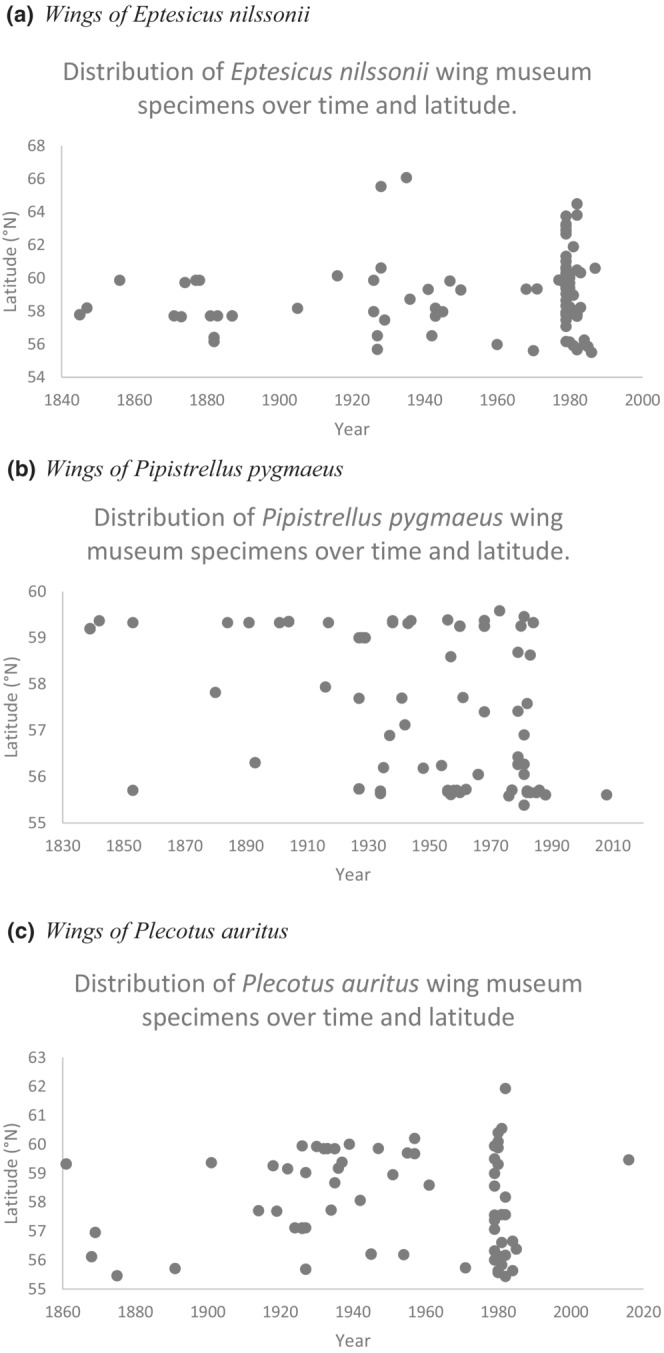
Spatial and temporal distribution of wing museum specimen collections showing the date of collection against the latitude of collection place in Sweden for three bat species (a–c)

Cranium specimens were dry with all fur and soft tissues removed whereas wing specimens were entire bats preserved in alcohol. Preservation in alcohol can cause desiccation of tissues but does not decalcify bones and this is why this method of preservation is often used for bone collection (Shields & Carlson, 1996). Little research has been conducted into the effects of alcohol preservation on bones, and as a precaution, we only measured wing specimens preserved in alcohol. In total, we measured 464 specimens, which is the entire Swedish collection of undamaged bat crania and wings available. They also had complete collection metadata including accurate geographical location and date of collection. In total, we measured 135 crania specimens (48 *Eptesicus nilssonii*, 43 *Pipistrellus pygmaeus*, and 44 *Plecotus auritus*) and wings of 329 specimens (96 *E. nilssonii*, 149 *P. pygmaeus*, and 84 *P. auritus*). Crania specimens dated from 1840–2014 whilst wing specimens were collected from 1839–2016 (Wood & Cousins, 2022); see Appendix A, Figures A1 and A2 for plots showing the spatial and temporal distribution of specimens for each species. There is no precise way to age bats, but similar to Tomassini et al. (2014), we only used specimens with full adult teeth with slight or moderate wear to the molars.

**FIGURE 2:**
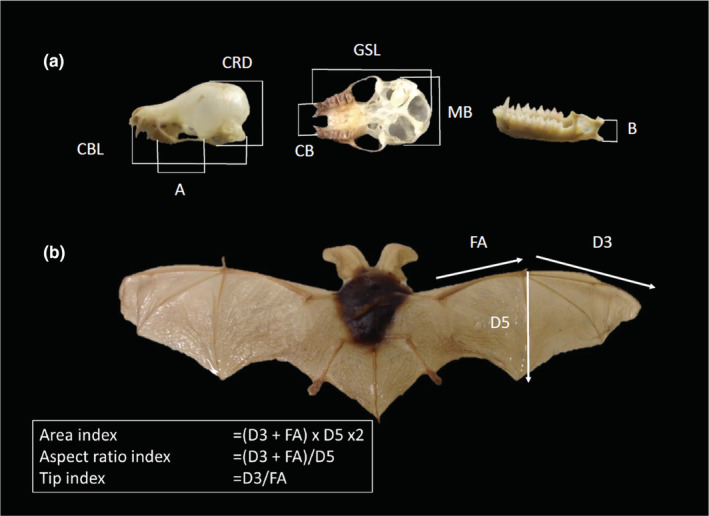
Measurements taken from the museum specimens of bats within this study. (a) Cranial and jaw measurements. A, length from the cranio‐mandibular joint to the most anterior point of origin of the masseter muscle; B, length from the cranio‐mandibular joint to the insertion of the masseter muscle at the bottom of the angular process; CB, canine breadth; CBL, condylobasal length; CRD, cranial depth; GSL, Greatest skull length; MB, mastoid breadth. (b) Wing morphological measurements. D3, digit three, from wrist to tip of wing; D5, digit five; FA, forearm length. We calculated three indices from the wing measurements: area index, aspect ratio index, and tip index. Photos: Heather Wood

We measured the cranial parameters following a similar method used by Tomassini et al. (2014) and previously adapted by Barlow et al. (1997). We modified this method by replacing the incisor measurement with the canine breadth (CB) measurement, as we found it difficult to accurately replicate this measurement on our specimens. We measured seven parameters of skulls: greatest skull length (GSL), mastoid breadth (MB), condylobasal length (CBL), cranial depth (CRD), canine breadth (CB), length from the cranio‐mandibular joint to the most anterior point of origin of the masseter muscle (A) and length from the cranio‐mandibular joint to the insertion of the masseter muscle at the bottom of the angular process (B) (Figure 2a).

Wing measurements of live bats typically involve three parameters: wing loading, aspect ratio, and tip shape index (Norberg, 1994). In order to calculate wing loading, the live body weight of the bat is required, and for aspect ratio and tip shape index, the surface area of the wing is required. Both these parameters are difficult to obtain from museum specimens because the live animal was often not weighed and the wing membrane can be damaged or difficult to fully stretch out in preserved specimens. To overcome this issue Findley et al. (1972) devised an alternative method to obtain similar data for museum specimens. They created three new parameters using measurements of the forearm and the third and fifth digits: wing area index, aspect ratio index, and tip index. Both wing loading and aspect ratio were shown to strongly correlate with the wing area index and aspect ratio index (Findley et al., 1972) and are therefore considered a good proxy for wing loading. Therefore, for each specimen, we measured four variables: forearm length and the three calculated parameters (wing area index, aspect ratio index, and tip index) as shown in Figure 2b. All wing measurements were taken on the right side (dorsal view) using Mitutoyo Absolute Digimatic Calipers, Series 500. Measurements were taken twice and averaged and only accepted if they were replicable within 0.05 mm. Otherwise, two new measurements were taken.

### 2.2 Land‐use data

Land‐use data were used as a proxy for resource availability, both habitat and prey availability in this study. We used different types of land‐use data depending on the collection date of the specimen. For modern species collected after 1978, we used the National Land Cover Data (Swedish: Marktäckedata [Swedish Environmental Protection Agency, 2020]) to extract land use in a landscape within a 2 km radius around the specimen. This buffer zone is considered appropriate as most temperate insectivorous bats typically have home ranges below 5 km (Altringham & Kerth, 2016). We reclassified the land use into three categories: Forest, Open, and Water, and areas with dense buildings or hard surfaces were categorized as “no data.”

Where specimens were collected between 1935 and 1978, we used the Economic Map series (Swedish: *Ekonomiska kartan*). These maps are monochrome orthophotos where arable fields are colored yellow and built surfaces in black. We implemented a rapid digitization method using the “HistMapR” package (Auffret et al., 2017) in R (R Core Team, 2019). This results in a simple but efficient classification of land use into three classes: arable, open land, and forest in a GIS format at a 1 m resolution. Domestic gardens were included in the arable land. Buildings themselves were not included, and no data on the area of built‐up environments are available for these time periods. We then reclassified the maps using arcgis (Esri, 2011) so they had only two categories: open (including arable, domestic gardens) and forest. The freshwater areas are difficult to digitize due to their color overlap with other land classes and instead were extracted afterwards using the present day land‐use maps (Lantmateriet, 2019) with the function gdal_rasterize in the package “gdalUtils” (Greenberg & Mattiuzzi, 2015). We then resampled the rasters to 5 m resolution using gdal_warp in the package “gdalUtils” and used gdal_warp to extract the land use.

**FIGURE B1:**
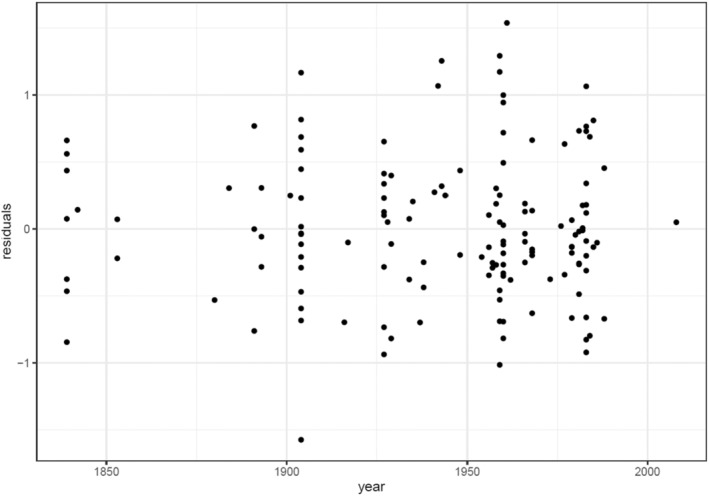
Plot of model residuals versus year for the forearm length of *Pipistrellus pygmaeus* mixed model

For the specimens collected prior to 1938, we used historic national statistics to model land use for 1865, 1900, 1920, 1944, and 1952 (Statistiska centralbyrån, 1868, 1869, 1900, 1920, 1944, 1952). In three cases data were unavailable for 1865 and we instead took the nearest available data—1869. Then, we assigned each specimen to the nearest time period. This method is similar to the one developed by Pasanen‐Mortensen et al. (2017) and uses land‐use data per district (Swedish: härad) to calculate the proportion of open and forest land (excluding water). To calculate the amount of open land in each 2 km radius landscape we identified which district the bat specimen was collected in. From the statistic tables, we calculated the percentage of open area in the district (gardens, arable, and meadows). The area of water is not included in the historic statistics thus we corrected this by using the present day water area extracted from the National Land cover Data (Swedish: Martäckedata [Swedish Environmental Protection Agency, 2020]). Any remaining area within the landscape was classified as forest. We acknowledge that using historic land‐use data from three different geographical sources is challenging, but in order to obtain land‐use data across our time period, we used all the data available. The two map data sources use aerial photography and satellite imagery as their primary data source and are reasonably comparable to each other. Furthermore, we verified the accuracy of our land use calculated by statistics with that extracted by manual map digitization and obtained an accuracy of 78%. This is similar to the 80%–90% accuracy achieved by HistMapR (Auffret et al., 2017). As such we are confident that our land‐use data are appropriate for this study. We also checked the residuals of our models against year and land‐use data type and found no effect on our model performance (see Appendix B, Figure B1).

### 2.3 Climate data

We used 0.5° gridded daily mean temperature data from the CRU database to calculate the summer average monthly temperature (Harris et al., 2014). The database covers the time period from 1901 so we used weather station data from the Swedish Meteorological Institute for the earlier time steps (SMHI, 2019). In total, we used data from 12 weather stations across Sweden for the 75 specimens that were collected pre‐1901. We selected the nearest weather station to the specimen along with the earliest weather recording corresponding to the bat collection date. Preliminary analysis of the climate data shows that temperature did not increase across our collection sites during our study period. We therefore will use the spatial variation in temperature as a space for time analysis to predict how rising temperatures in the future may affect bat morphology. Examination of the residuals of the model of temperature against year whilst controlling for latitude as a random variable shows that the residual variance of temperature during our study period is stable and as such suitable for space for time analysis. We also examined if the temporal temperature change in Sweden is equivalent to the spatial variation in temperature in our data set. We examined the range of summer monthly average temperatures in our spatial data set and compared to those summer monthly average temperatures in three locations in Sweden (one southern, one central, and one northern location) at two time points: 1862 and 2017 (and therefore covering most of our study period). We found that the range of temperatures that we find spatially (7.7 to 16.9°C) covers completely the temporal range found at these three latitudes (from 10.83°C in 1862 in the north to 15.9°C in 2017 in the south). Furthermore, our maximum temperature of 16.9°C actually exceeds the 2017 temperatures in the south (15.9°C), central (15.2°C), and northern (11.5°C) Sweden by 1, 1.7, and 5.4°C. Therefore, our spatial temperature range covers temperature rises that could be expected with future climate change.

### 2.4 Statistical analysis

As is common with many morphological data sets (Clavel et al., 2014), the cranial data set had a proportion of missing values for certain cranial measurements. In our cranial data set, 17.8% of the data was missing due to damage to the skulls. Many imputation methods are available to handle missing data and their effectiveness depends on both the rate of missingness but also the proportion of specimens to variables. Our ratio was 19.3 (135 specimens/7 variables) and is considerably higher than the full data set ratio (226/26 = 8.7) used by Clavel et al. (2014). They conclude that the Amelia imputation method works best with this ratio up to a missing value rate of 25%. Hence we used the package “Amelia” (Honaker et al., 2011) in R to impute the cranial data set. We ran 5 imputations of the data set and carried out diagnostic checks on the effectiveness of the imputation by checking density plots observed against imputed data and checking for overimputation via the overimpute command in the Amelia package. This led to us removing CB from the *P. pygmaeus* data and MB from the *P. auritus* data as they were overimputed whereas all seven variables were retained for *E. nilssonii*. As the cranial variables are all highly correlated we carried out a data reduction using a Principal Components Analysis (PCA) using covariance matrices in the “prcomp” package in R (R Core Team, 2019). Variables were not transformed into logs, as they met the linearity condition via visual inspection of plots. To ease our interpretation of the effect of PC1 and PC2, we reversed the signs of the PC1 and PC2 scores. This was because increasing PC1 and PC2 tended to be related to decreasing skull and jaw parameters. As the sign of PC scores is arbitrary this does not affect the variance explained by the component (Livingstone, 2009).

We created linear mixed models (R function: lme, “nlme” package) to test the effect of temperature and resource availability variables on our six response variables: PC1, PC2, forearm length, aspect ratio index, wing area index, and tip index. Each model included one climatic variable (summer average temperature), and the latitude of the specimens. Specimens with the same collection number were collected at the same locality and at the same time. Therefore, to control for spatial and temporal autocorrelation, the collection number of the specimen was used as a random factor in the models. We used one resource availability variable, represented by the proportion of forest in each landscape. Proportion of forest accounts for both prey and habitat variability and is a known strong predictor of bat occurrence for our chosen three bat species (Ekman & de Jong, 1996). The predictor variables had low pairwise correlation coefficients (*r* < .5) except in the case of temperature and latitude (*r* = .67), but still, this does not exceed the threshold of .7 as suggested by Dormann et al. (2013) and so both variables were retained in the models.

If either temperature or forest cover had a significant relationship with a morphological variable then the year of collection was included, first additively and then as an interaction to test the effect over time. We retained the year of collection if it significantly improved the model. We tested for sexual dimorphism of skull or wing parameters using *t*‐tests, and if sexual dimorphism was detected, we included sex in the model. *P. pygmaeus* was sexually dimorphic in regards to PC2, forearm length, and wing area index, and *E. nilssonii* was sexually dimorphic in relation to PC1 and wing area index. No sexual dimorphism was detected in *P. auritus*. This resulted in six full models for the cranial data set and 12 full models for the wing data set (see Appendix C). We standardized the coefficients by dividing each variable by its standard deviation using the scale function in R to allow for a direct comparison of effect sizes in each model (Gelman, 2008). We also ran simple linear models to assess the trends in climate and landscape variables throughout our study period (R function: lm, “stats” package). We validated all models by visually examining the residual plots and marginal and conditional R‐squared values are reported for each.

## 3 RESULTS

During our 180‐year study period, forest cover increased within the landscapes surrounding where the specimens were collected (Estimate = 0.001, *p* < .001); the same trend was found with increasing latitude (Estimate = 0.021, *p* < .001). As for temperature, it decreased with latitude at the collection localities (Estimate = −0.385, *p* < .001), and there was no evidence that temperature increased over time at our collection points (Estimate = 0.001, *p* = .880).

The first (PC1), second (PC2), and third (PC3) principal components for *Pipistrellus pygmaeus* explained 50.3%, 21.8%, and 12.4% of the variation in skull size whereas in *Plecotus auritus* PC1, PC2 and PC3 explained 56.8%, 18.3%, 12.1% of cranial variation with corresponding values of 48.0%, 17.9% and 13.5% in *E. nilssonii*. The variables with the strongest weighting on the first principal component for all three species were CBL and GSL; these are two measurements indicating the total length of the skull. PC2 differentiated B, A and MB in *P. pygmaeus*, B, CRD and A in *P. auritus*, and B, CRD and MB in *Eptesicus nilssonii* (Appendix D). Both B and A are measures of the jaw relating to the moment arm of the masseter and gape (Herring & Herring, 1974) whereas MB and CRD are the breadth and depth of the skull and are related to muscle attachment for the jaw with carnivores having greater breadth and depth for eating larger prey items (Van Cakenberghe et al., 2002). We used the scores of PC1 and PC2 as response variables in further analysis, but as PC3 did not clearly differentiate other skull variables from those represented by PC1 and PC2, it was excluded.

Mean summer temperature did not have a significant effect on cranial or jaw size, as represented by PC1 and PC2 scores (Table 1). However, the jaws (specifically the moment arm of the masseter) of *P. auritus* became larger over time (Estimate = 0.307, *p* < .01) while those of *P. pygmaeus* became smaller (Estimate = −0.762, *p* = .020) as shown by increasing and decreasing PC2 scores (Table 1 and Appendix E, Figures E1a,b).

**TABLE 1:** Linear model coefficients of the influence of forest cover and temperature (summer monthly average) on bat cranial size as represented by the PC1 and PC2 scores.

Variable	*Pipistrellus pygmaeus*						*Plecotus auritus*						*Eptesicus nilssonii*					
	PC1			PC2			PC1			PC2			PC1			PC2		
	Estimate	*t*	*p*	Estimate	*t*	*p*	Estimate	*t*	*p*	Estimate	*t*	*p*	Estimate	*t*	*p*	Estimate	*t*	*p*
Forest cover (%)	−0.598	−2.052	.070	−0.134	−0.612	.560	−0.014	−0.058	.954	−0.140	−1.252	.218	0.492	1.747	.095	−0.064	−0.354	.725
Temperature (°C)	0.052	0.166	.869	0.066	0.302	.766	0.235	0.706	.484	0.285	1.801	.080	−0.468	−1.018	.320	0.269	0.950	.349
Latitude	0.347	1.179	.268	0.020	0.093	.928	0.096	0.312	.757	−0.043	−0.298	.767	−0.593	−1.138	.284	0.051	0.159	.877
Year				**−0.762**	**−2.525**	.020*				**0.307**	**2.917**	<.01**						
Sex‐Male				−0.574	−1.557	.163							**−1.939**	**−3.028**	.014*			
Marginal *R* ^2^	.115			.363			.014			.333			.361			.064		
Conditional *R* ^2^	.365			.363			.014			.353			.397			.567		

*Note*: The PC scores were based on measurements of 135 bat museum specimens collected in Sweden between 1840–2014. Values are shown from the full model. Significance levels given: ***<0.001, **<0.01, *<0.05,< 0.1.

Bold values are significance levels: ***<0.001, **<0.01, *<0.05, < 0.1

The data revealed evidence for interacting effects on bat wing measurements in one species, (Table 2) where forest cover and temperature had a strong interactive effect on the tip index of *P. pygmaeus* (Estimate = 0.025, *p* < .001). This interaction shows that the relationship between declining tip index with increasing forest cover becomes positive after approximately 14.5°C and that the tip index then increases in relation to forest cover (See Appendix E, Figure E2). Temperature also had a positive correlation with the tip index in *P. auritus* (Estimate = 0.015, *p* = .037), but as this model had a low *R* ^2^ it is important not to overinterpret this result. *E. nilssonii* tip index was positively correlated with forest cover (Estimate = 0.021, *p* = .020) and negatively with year (Estimate = −0.026, *p* < .01). As for the aspect ratio index, we found a lower aspect ratio in *P. pygmaeus* in more forested landscapes (Estimate = −0.019, *p* < .01). We also found a negative correlation between aspect ratio and temperature in *P. auritus* (Estimate = −0.025, *p* = .028), but this model had a low marginal *R* ^2^. A similar declining aspect ratio was found over time in *E. nilssonii* (Estimate = −0.016, *p* = .031). In parallel, the forearm length of *E. nilssonii* declined with increasing forest cover (Estimate = −0.344, *p* = .031). A similar trend was observed in *P. auritus* except that the decline in the forearm was correlated with the year (Estimate = −0.304, *p* = .029). Wing area index declined over time in *E. nilssonii* (Estimate = −2.158, *p* = .048) and increased with latitude in *P. auritus* (Estimate = 2.023, *p* = .046) (Appendix E, Figure E1).

**TABLE 2:** Linear model coefficients of the influence of forest and temperature (summer monthly average) on bat wing morphology based on measurements of 329 bat museum specimens collected in Sweden between 1839–2016.

(a) Linear model results for *Pipistrellus pygmaeus*. Based on wing measurements of 149 museum specimens												
Variable	*Pipistrellus pygmaeus*											
	Aspect ratio index			Tip index			Wing area index			Forearm length		
	Estimate	*t*	*p*	Estimate	*t*	*p*	Estimate	*t*	*p*	Estimate	*t*	*p*
Forest cover (%)	**−0.019**	**−2.871**	<.01**	**−0.016**	**−2.541**	.013*	0.632	1.099	.275	0.105	0.757	.451
Temperature (°C)	0.008	0.904	.369	**0.035**	**4.148**	<.001***	0.749	0.938	.352	−0.013	−0.068	.946
Latitude	−0.008	−0.940	.350	−0.006	−0.780	.438	−0.190	−0.265	.792	−0.020	−0.115	.909
Sex‐Male							−0.702	−1.052	.296	−0.106	−0.728	.469
Forest cover × Temperature				**0.025**	**3.553**	<.001***						
Marginal *R* ^2^		.153			.258			.258			.013	
Conditional *R* ^2^		.515			.486			.486			.617	

(b) Linear model results for *Plecotus auritus*. Based on wing measurements of 84 museum specimens												
Variable	*Plecotus auritus*											
	Aspect ratio index			Tip index			Wing area index			Forearm length		
	Estimate	*t*	*p*	Estimate	*t*	*p*	Estimate	*t*	*p*	Estimate	*t*	*p*
Forest cover (%)	−0.007	−0.859	.403	0.005	0.893	.384	−0.507	−0.582	.568	−0.266	−1.794	.091
Temperature (°C)	**−0.025**	**−2.257**	.028*	**0.015**	**2.134**	.037*	1.025	1.106	.273	−0.190	−1.009	.317
Latitude	−0.007	−0.723	.479	−0.001	−0.158	.876	**2.023**	**2.169**	.046*	0.299	1.885	.077
Year							−1.590	−1.978	.053	**−0.304**	**−2.235**	.029*
Sex‐Male							−2.084	−1.563	.138			
Marginal *R* ^2^	.085			.081			.164			.201		
Conditional *R* ^2^	.650			.081			.369			.350		

(c) Linear model results for *Eptesicus nilssonii*. Based on wing measurements of 96 museum specimens												
Variable	*Eptesicus nilssonii*											
	Aspect ratio index			Tip index			Wing area index			Forearm length		
	Estimate	*t*	*p*	Estimate	*t*	*p*	Estimate	*t*	*p*	Estimate	*t*	*p*
Forest cover (%)	−0.005	−0.621	.536	**0.021**	**2.367**	.020*	0.739	0.704	.484	**−0.344**	**−2.194**	.031*
Temperature (°C)	0.015	1.681	.097	0.010	0.929	.356	1.006	0.784	.436	0.195	0.877	.383
Year	**−0.016**	**−2.190**	.031*	**−0.026**	**−3.001**	<.01**	**−2.158**	**−2.009**	.048*			
Latitude	0.017	1.776	.114	0.015	1.348	.215	1.023	0.784	.459	0.106	0.460	.658
Sex‐Male							−4.042	−2.184	.065			
Marginal *R* ^2^	.116			.113			.123			.069		
Conditional *R* ^2^	.116			.113			.259			.508		

*Note*: Values are shown from the full model.

Bold values are significance levels: ***<0.001, **<0.01, *<0.05, < 0.1

## 4 DISCUSSION

We found differences in bat morphology due to geographic variability in temperature and land‐use factors over 180 years and across a 1200 km gradient in Sweden. We propose that the main mechanisms behind this variation are due to selective pressures of thermal regulation and resource availability, both prey and habitat.

Our finding of increasing forest over the duration of our study period corresponds well with the national statistics on forest cover where the standing volume of trees in forests has doubled in the last 100 years (Roberge et al., 2020). By contrast, we did not find evidence of increasing summer temperatures during the same period at our study sites, but this is unsurprising as we do not have a repeated annual time series of climate data at each of our study sites. However, temperatures in northern Sweden have risen by 1.5–2°C between 1959 and 2018 (Stoessel et al., 2022). This study analyzed changes in temperature in every grid cell over northern Sweden using the same data set as we used, but crucially, they had a complete time series of data for every year and can truly validate that climate change has occurred. We would like to emphasize that it is still important to explore how spatial variation in temperature and forest cover affects morphology as it may give insights into how species may respond to future changes.

We explored the effect of mean summer temperature and resource availability (namely forest cover) on the morphology of three bat species in different foraging guilds based on 464 museum specimens. These bat guilds overlap slightly along a spectrum of forest to open land specialism with *Plecotus auritus* being more forest‐dependent; *Pipistrellus pygmaeus* is an interface specialist; and *Eptesicus nilssonii* is more adapted to open habitats (De Jong et al., 2020). Morphological parameters relating to body size did not decrease with increasing temperatures nor did appendage size increase with temperature, as stated in our first hypothesis. Wing area index, on the contrary, did increase with latitude in *P. auritus*. We also found increasing jaw size in *P. auritus* and decreasing jaw size in *P. pygmaeus* over time. Regarding wing functional traits, *P. pygmaeus* wings were shorter and broader and less pointed in more forested landscapes and therefore support our second hypothesis. Similarly, *E. nilssonii* wings were shorter, broader, and less pointed, with reduced wing area over time and had shorter forearms, and were more pointed with increasing forest. Finally, we can confirm our third hypothesis that forest and temperature interact antagonistically causing variation in tip index in different directions, in *P. pygmaeus*.

Our results provide no support for Bergmann's and Allen's rule in Swedish bat populations. Despite the known relationship between higher temperature and body size suggesting that a warming climate will cause body sizes to decrease over time, so far there is little evidence that these changes have occurred yet (Meiri et al., 2003). Whereas Allen's rule has been confirmed for a broad geographical range of taxa, but studies are still predominately focused on birds (Ryding et al., 2021).

However, accurately testing these rules previously has been complicated by the use of the year as a proxy for climatic variables. This makes disentangling the effects of climate and land use on morphology difficult, as land use also varies over time. These studies may have also missed key interactions and particularly antagonistic ones that may drive responses in opposite directions (Oliver & Morecroft, 2014). Among studies that used actual climate data, many found an effect of rising temperatures on body size, but the direction of the effect is inconsistent (Yom‐tov et al., 2008, 2010; Yom‐Tov & Yom‐Tov, 2004). As for land‐use change data, attempts have been made to incorporate this into morphological studies. This is difficult, as most often, the mapping data does not adequately cover the timescale of interest (Yue et al., 2020), or it is represented by other historical data. For example, historical data on street lighting was used as a proxy for urbanization and was strongly correlated with increasing cranial size in bats due to higher resource availability (Tomassini et al., 2014). By combining both climate and land‐use data our study offers the unique opportunity to explore the interactions of these temperature and land‐use variables on bat morphology.

In our study we found temporal changes in skull measurements but not in relation to climatic variables. Specifically, jaw size increased in *P. auritus* over time and decreased in *P. pygmaeus*. No other variables were significant, but there was a trend of decreasing jaw sizes with increasing forest cover in *P. pygmaeus*. Similar to our observations in *P. auritus*, larger jaw sizes (larger gape, masseter muscle, and height of coronoid process) were found in a tropical bat species that broadened its primarily moth‐based diet to include beetles (Jacobs, 1996). Similarly, *P. auritus* is a moth specialist and potentially this result is indicating a shift to larger prey items, e.g., Coleoptera (beetles), which is their second preferred prey after moths (Rydell, 1989). However, without more detailed information about the regional variation of *P. auritus* diet in Sweden, it is difficult to extrapolate our findings of variation in jaw measurements to dietary differences due to prey availability in more forested landscapes. As for the decreasing jaw size of *P. pygmaeus*, we are not aware of any other studies finding similar results. Potentially we are observing an effect of foraging habitat choice with more forested landscapes supporting a higher number of smaller prey items such as midges. It is known that soft‐bodied Nemoteceran diptera are the main components of *P. pygmaeus* diet (Barlow et al., 1997). Coniferous forest habitats support high abundances of Nemoteceran diptera, e.g., midges, and *P. pygmaeus* will preferentially seek out the coniferous forest for foraging (Kirkpatrick et al., 2018). However, we mirror the sentiments of Salinas‐Ramos, Agnelli, Bosso, Ancillotto, Sánchez‐Cordero, et al. (2021), who found changes in bat jaw morphology along a latitudinal gradient and hypothesized this may be due to a key variable relating to diet being absent from the data. We think these findings merit further investigation of both jaw size and diet variability of bats across their natural ranges to possibly unravel additional effects of temperature and resource availability on jaw morphology.

Our wing data revealed declines in forearm length in *E. nilssonii* in relation to forest cover and for *P. auritus* over time. Forearms of *P. auritus* also showed a tendency to decline with increasing forest cover, but this result was not significant. Declining forearm lengths in more forested landscapes could be due to a need to move through a more cluttered landscape as bats with shorter forearms tend to have low aspect ratios adapted for flight in forested environments (Norberg & Rayner, 1987). Both the cessation of forest grazing prior to 1940 (Jensen, 2011) and an increase in plantation forestry dominated by Scot's pine (*Pinus sylvestris*) and Norway spruce (*Picea abies*) have led to denser forests (Jansson, 2011) and more monocultures in the present day. Denser stands of plantation forestry are less suitable for most bat species, but our findings show that bat wings have the potential to adapt to increases in forest habitat availability. Whether this response is due to phenotypic plasticity or genetic evolution merits further research.

We also show that *P. pygmaeus* wings were shorter, broader, and less pointed with increasing forest cover and therefore more adapted to flying through this habitat. *E. nilssonii* wings also became shorter, broader, and less pointed with smaller wing area over time but conversely more pointed in relation to forest cover. It is unclear what processes are driving the variation in wing morphology in *E. nilssonii* but possibly more subtle habitat preferences not captured by our models could be driving these changes. Despite this uncertainty, the changes in wing functional traits are interesting and merit further investigation. Particularly, it is crucial to understand how forest structure dynamics (e.g., temporal changes in tree composition and density) influences bat morphology; rather than only forest cover per se. Many studies have shown interspecific variability in bat wing morphology (Findley et al., 1972; Jones et al., 2003; Norberg & Rayner, 1987) and a few have shown that this variation also occurs intraspecifically due to the influence of climatic variables (Burnett, 1983; Jacobs, 1996). However, this is to our knowledge the first study to show that the amount of available habitat, namely forest cover, can be responsible for intraspecific variability in wing functional traits. This shows the flexibility of *P. pygmaeus* to adapt to more forested landscapes, with short, broader, and less pointed wings, but it is worth noting that a low aspect ratio index has also been linked to higher extinction risk in bats due to their specialization in forested habitats, which are declining globally (Jones et al., 2003).

Notably, we found an antagonistic interaction between forest and temperature on the tip index in P. *pygmaeus*. Wing tip initially became less pointed with forest cover, but over a threshold temperature of 14.5°C, their wings are shifting to become more pointed. It is known that a high aspect ratio and wing pointedness increases efficiency (Norberg, 1994) and speed of flight (Findley et al., 1972) in bats, and migratory birds with more pointed wings have lower flight costs (Bowlin & Wikelski, 2008). What is less well‐studied is the effect of higher temperatures on energy use and speed during flight. One study suggests that the amount of energy used during flight is relatively constant across temperature gradients, but they also highlight that under higher temperatures the air density is lower and this increases energy expenditure (Rubalcaba et al., 2022). This could favor pointed wings that are more efficient under warmer temperatures. This interactive effect on the tip index also highlights that there could be a trade‐off between adapting energetically with pointed wings versus adapting to increased forest habitat availability in the landscape with less pointed wings.

## 5 CONCLUSION

Our data shows the value of using the unique time series of morphological data offered by museum collections to assess the effects of temperature and resource availability on biodiversity. We demonstrate the importance of including both climatic and land‐use variables when assessing trends in bat morphology and exploring their potential interactions and can show that geographical variability in these drivers is linked to differences in bat morphology. This morphological variation is likely due to the selective pressures of thermal regulation and resource availability. It is worth noting that our specimens are limited to Sweden, albeit along a 1200 km gradient, and the three species studied have geographic ranges that cover large parts of Europe and Asia. As such further studies are required to understand whether the trends we find along this gradient are consistent over these species' ranges. As previously stated, bats are long‐lived organisms and detect changes over time due to climate and land‐use factors may be more difficult compared with short‐living organisms. Furthermore, other factors such as evolutionary processes or genetics play a larger role in determining wing functional traits. Regardless, these findings provide an insight into the potential future changes in morphology. Encouragingly all three species have shown an ability to adapt their body size and functional traits to different conditions and could demonstrate their potential to overcome the future negative impacts of climate and land‐use change.

## AUTHOR CONTRIBUTIONS

**Heather Wood:** Conceptualization (equal); data curation (lead); formal analysis (lead); investigation (lead); methodology (equal); validation (lead); visualization (lead); writing – original draft (lead); writing – review and editing (equal). **Sara A. O. Cousins:** Conceptualization (equal); formal analysis (supporting); investigation (supporting); methodology (equal); project administration (lead); supervision (lead); visualization (supporting); writing – original draft (supporting); writing – review and editing (equal).

## CONFLICT OF INTEREST

The authors declare no conflict of interest.

## Data Availability

All morphological data are available via the Bolin Centre for Climate Research Database at https://doi.org/10.17043/wood‐2022‐bats‐1.

## APPENDIX B

### Model residuals to explore the effect of using different land‐use data sets

Here we present our data exploration of model residuals to investigate the effect of using three different land‐use data sets. First, we plotted the model residuals versus the land‐use data type for each model. In some cases, the residual spread was lower for one data set versus the other two. In the wing data set this was the case for the Modern land‐use data set and this is simply because the number of modern specimens was low and so we had less residual variance. To check how this affected our models we re‐ran them minus the modern specimens and this had no effect on the trends in our models. We also plotted the residuals against collection year and found no discernible pattern other than there are fewer points at either end of the spectrum of time and that is simply due to there being fewer very old and very modern specimens. See Figure B1 below for an example of this plot.

The pattern shown in Figure B1 was the same for all wing models. In terms of the cranial data set, the residual variance was sometimes lower in one category compared with the other two, and this again was due to sample size. We re‐ran each model minus the data from the land use category with the lower residual variance and again found the exact same trends in the reduced models.

As such we are confident that the three different land‐use data sets are not influencing our results.

## APPENDIX C

### Full models from the mixed linear regression

Crania models

*Models 1 & 2 Pipistrellus pygmaeus*

lme (PC1Score ~ ForestCover + SummerAverageTemperature + Latitude + random = ~1|CollectionNumber)

lme (PC2Score ~ ForestCover + SummerAverageTemperature + Latitude + Sex + Year + random = ~1|CollectionNumber)

*Models 3 & 4 Plecotus auritus*

lme (PC1Score ~ ForestCover + SummerAverageTemperature + Latitude + random = ~1|CollectionNumber)

lme (PC2Score ~ ForestCover + SummerAverageTemperature + Latitude + Year + random = ~1|CollectionNumber)

*Models 5 & 6 Eptesicus nilssonii*

lme (PC1Score ~ ForestCover + SummerAverageTemperature + Latitude + Sex + random = ~1|CollectionNumber)

lme (PC2Score ~ ForestCover + SummerAverageTemperature + Latitude + random = ~1|CollectionNumber)

Wing models

*Models 7–10 Pipistrellus pygmaeus*

lme (ForearmLength ~ ForestCover + SummerAverageTemperature + Latitude + Sex + random = ~1|CollectionNumber)

lme (AspectRatioIndex ~ ForestCover + SummerAverageTemperature + Latitude + random = ~1|CollectionNumber)

lme (WingAreaIndex ~ ForestCover + SummerAverageTemperature + Latitude + Sex + random = ~1|CollectionNumber)

lme (TipIndex ~ ForestCover * SummerAverageTemperature + Latitude + random = ~1|CollectionNumber)

*Models 11–14 Plecotus auritus*

lme (ForearmLength ~ ForestCover + SummerAverageTemperature + Latitude + random = ~1|CollectionNumber)

lme (AspectRatioIndex ~ ForestCover + SummerAverageTemperature + Latitude + random = ~1|CollectionNumber)

lme (WingAreaIndex ~ SummerAverageTemperature * Year + ForestCover + Latitude + random = ~1|CollectionNumber)

lme (TipIndex ~ ForestCover + SummerAverageTemperature + Latitude + random = ~1|CollectionNumber)

*Models 15–18 Eptesicus nilssonii*

lme (ForearmLength ~ ForestCover + SummerAverageTemperature + Latitude + random = ~1|CollectionNumber)

lme (AspectRatioIndex ~ ForestCover + SummerAverageTemperature + Latitude + random = ~1|CollectionNumber)

lme (WingAreaIndex ~ ForestCover + SummerAverageTemperature + Latitude + Sex + random = ~1|CollectionNumber)

lme (TipIndex ~ ForestCover + SummerAverageTemperature + Latitude + random = ~1|CollectionNumber)

## APPENDIX D

- Weightings for the first three components of Principle Components Analysis of six measurements from the crania of *Pipistrellus pygmaeus* from 43 individuals collected in Sweden. Six variables were retained after checking for overimputation: greatest skull length (GSL), condylobasal length (CBL), cranial depth (CRD), mastoid breadth (MB), length from the cranio‐mandibular joint to the origin of the masseter muscle (A) and length from the cranio‐mandibular joint to the insertion of the masseter muscle at the bottom of the angular process (B).

**Table:**

	PC1	PC2	PC3
GSL	−0.49845	0.303558	−0.2323
CBL	−0.51372	0.205345	−0.28508
CRD	−0.25447	0.562249	0.628975
MB	−0.40387	−0.23578	0.401977
A	−0.43436	−0.31595	−0.38409
B	−0.26666	−0.62781	0.400056

b Weightings for the first three components of Principle Components Analysis of six measurements from the crania of *Plecotus auritus* from 44 individuals collected in Sweden. Six variables were retained after checking for overimputation: greatest skull length (GSL), condylobasal length (CBL), cranial depth (CRD), canine breadth (CB), length from the cranio‐mandibular joint to the origin of the masseter muscle (A) and length from the cranio‐mandibular joint to the insertion of the masseter muscle at the bottom of the angular process (B)

**Table:**

	PC1	PC2	PC3
GSL	−0.48282	0.324856	−0.08697
CBL	−0.48657	0.29798	−0.09666
CRD	−0.39246	−0.48558	0.208327
Canine	−0.37119	0.461532	0.329367
A	−0.3372	−0.29295	−0.7996
B	−0.35302	−0.52063	0.438016

c Weightings for the first three components of Principle Components Analysis of seven measurements from the crania of *Eptiscus nilssonii* from 48 individuals collected in Sweden. All seven crania variables were retained after checking for overimputation: greatest skull length (GSL), mastoid breadth (MB), condylobasal length (CBL), cranial depth (CRD), canine breadth (CB), length from the cranio‐mandibular joint to the origin of the masseter muscle (A) and length from the cranio‐mandibular joint to the insertion of the masseter muscle at the bottom of the angular process (B).

**Table:**

	PC1	PC2	PC3
GSL	−0.50549	0.201663	−0.06283
CBL	−0.47013	0.393925	−0.03829
CRD	−0.2294	−0.51896	0.543352
MB	−0.31906	−0.36413	−0.41886
Canine	−0.32496	0.185883	0.656081
A	−0.45352	0.071453	−0.2912
B	−0.24033	−0.60216	−0.0931

## APPENDIX E

Response curves from our data analysis where we used a combination of high spatial and temporal resolution climate and land‐use data to assess how bat cranial size and wing functional traits respond to changing temperatures and forest cover over 180 years along a 1200 km latitudinal gradient.

E1: Predicted response curves showing the relationship between predictor and response variables. Plots (a and b) show the crania response curves and (c‐l) show the wing response curves. No response curves are provided if sex was the only significant variable in the model. All plots were produced using the visreg package in R (R Core Team, 2019). Regression lines and confidence intervals are given after accounting for slope estimates on median values of the other model variables.

(a) Relationship between Year and PC2 values of the crania variables of *Pipistrellus pygmaeus*. PC2 corresponds to increase in three jaw measurements: length from the cranio‐mandibular joint to the origin of the masseter muscle (A), length from the cranio‐mandibular joint to the insertion of the masseter muscle at the bottom of the angular process (B), and cranial depth (CRD).

(b) Relationship between Year and PC2 values of the crania variables of *Plecotus auritus*. PC2 corresponds to increase in three jaw measurements: length from the cranio‐mandibular joint to the origin of the masseter muscle (A), length from the cranio‐mandibular joint to the insertion of the masseter muscle at the bottom of the angular process (B), and cranial depth (CRD).

(c) Relationship between proportion of forest in the landscape (2 km radius from specimen) and aspect ratio of wings of *Pipistrellus pygmaeus*.

(d) Relationship between summer average temperature and aspect ratio of wings *Plecotus auritus*.

(e) Relationship between summer average temperature and tip index of wings of *Plecotus auritus*.

(f) Relationship between latitude of collection and wing area index of wings of *Plecotus auritus*.

(g) Relationship between year of collection and forearm length of *Plecotus auritus*.

(h) Relationship between the year of collection and Aspect Ratio of wings of *Eptesicus nilssonii*.

(i) Relationship between proportion of forest in the landscape (2 km radius from specimen) and Tip Index of wings of *Eptesicus nilssonii*.

(j) Relationship between year of collection and tip index of wings in *Eptesicus nilssonii*.

(k) Relationship between year of collection and wing area index of wings of *Eptesicus nilssonii*.

(l) Relationship between proportion of the forest in the landscape (2 km radius from specimen) and forearm length of *Eptesicus nilssonii*.

E2: Conditioning plots (Coplot a) show the effect of significant interactions of predictor variables on the wing response variables. Year was included as an interaction in the models if either temperature or proportion of forest was significant and in all models the interaction between forest and temperature was tested. If the interaction significantly improved the model (using ANOVA (model 1, model 2) in R), then the interaction term was retained and the plots are provided below. To read the coplots, read from bottom left to top right. Each overlapping bar at the top corresponds to the one coplot and should be read from left to right.

(a) Relationship between proportion of forest in the landscape (2 km radius from specimen) and the tip index of wings of *Pipistrellus pygmaeus* at varying summer average temperatures (°C).
